# Severe Varus Knee Deformity After Malunited Proximal Tibial Fracture Treated With Open-Wedge Distal Tuberosity Osteotomy: A Case Report

**DOI:** 10.7759/cureus.100245

**Published:** 2025-12-28

**Authors:** Shotaro Kawano, Toshihiro Seki, Eiichi Shiigi, Hiroshi Tanaka, Takashi Sakai

**Affiliations:** 1 Department of Orthopedic Surgery, Yamaguchi Prefectural Grand Medical Center, Hofu, JPN; 2 Department of Orthopedic Surgery, Yamaguchi University Graduate School of Medicine, Ube, JPN

**Keywords:** autologous bone grafting, case report, leg length discrepancy, open-wedge distal tuberosity osteotomy, patellar height, severe varus knee deformity

## Abstract

Varus knee deformity following proximal tibial fracture malunion is uncommon and poses surgical challenges due to altered anatomy and large correction requirements. We report the case of a 54-year-old man with progressive right knee pain and severe varus deformity secondary to a proximal tibial fracture sustained in childhood. Radiographs revealed a femorotibial angle (FTA) of 188° and a medial proximal tibial angle (MPTA) of 68°. Preoperative standing full-length radiographs demonstrated a leg length discrepancy (LLD), with a spina-malleolar distance (SMD) of 84.3 cm on the affected side and 87.5 cm on the contralateral side.

Open-wedge distal tuberosity tibial osteotomy (OWDTO) with a 20° correction was performed. The osteotomy gap was filled with autologous iliac bone and β-tricalcium phosphate, and stable fixation was achieved with double plating. A concomitant medial meniscus posterior root tear was repaired arthroscopically. Postoperative alignment improved, with a weight-bearing line ratio (%WBL) of 59% and an MPTA of 88°, and bone union was achieved without complications. Postoperatively, the radiographic SMD improved to 86.5 cm, reducing the limb length discrepancy to approximately 1 cm.

The Caton-Deschamps index remained unchanged postoperatively, indicating that patellar height was preserved. At 12 months, the patient was pain-free and had returned to work. This case highlights the advantages of OWDTO in achieving large angular correction while minimizing the risk of patella baja and improving limb length symmetry. Additionally, the combination of autologous bone grafting and double plating provided sufficient stability and promoted favorable bone healing. OWDTO with autologous bone grafting and double plating is a valuable surgical option for severe post-traumatic varus knee deformities, allowing reliable correction, preservation of patellar height, improvement of LLD, and functional recovery.

## Introduction

Periarticular osteotomy around the knee is widely performed as an effective surgical procedure for the correction of varus deformity. Among these, proximal tibial osteotomy is the most common approach and can be broadly categorized into two types: open-wedge and closed-wedge techniques. Representative procedures include the open-wedge high tibial osteotomy (OWHTO) using a locking plate [1], the open-wedge distal tuberosity osteotomy (OWDTO) performed distal to the tibial tuberosity [2], and the hybrid closed-wedge high tibial osteotomy (HCWHTO), which combines a medial open wedge and a lateral closed wedge [3]. Selection of the appropriate osteotomy technique according to the individual deformity pattern and the patient's condition is essential for achieving optimal outcomes.

Severe post-traumatic varus malalignment requiring large angular correction is uncommon and often presents unique technical challenges. While OWHTO and HCWHTO are widely used, these techniques may have limitations, including the risk of patella baja, insufficient correction capacity, or undesired limb-length changes, in cases requiring substantial correction.

We report a rare case of severe varus knee deformity with leg length discrepancy (LLD) secondary to malunion following a proximal tibial fracture. The patient was successfully treated with OWDTO combined with autologous bone grafting. This case is of particular interest because OWDTO was selected to achieve substantial angular correction while simultaneously improving LLD and maintaining patellar height. Furthermore, autologous bone grafting was performed to prevent nonunion, and double-plate fixation was used to address potential instability due to the large opening gap, features that highlight the technical advantages of this procedure.

## Case presentation

A 54-year-old man sustained a right proximal tibial fracture in a traffic accident at the age of 11. He underwent two open reduction and internal fixation (ORIF) procedures at a local hospital. Although implant removal was subsequently performed, a residual varus deformity and gait disturbance persisted. He had been working at construction sites, but his right knee pain gradually worsened in recent years, leading to referral to our institution.

His medical history was notable only for an appendectomy, with no significant comorbidities. He had a history of smoking until the age of 30, but was currently a non-smoker. On physical examination, he demonstrated an antalgic gait. The right knee showed a range of motion (ROM) of 130° flexion and -15° extension. The spina-malleolar distance (SMD) was 80 cm on the right and 83 cm on the left. McMurray, Watson-Jones, Lachman, and pivot-shift tests were all negative.

**Figure 1:**
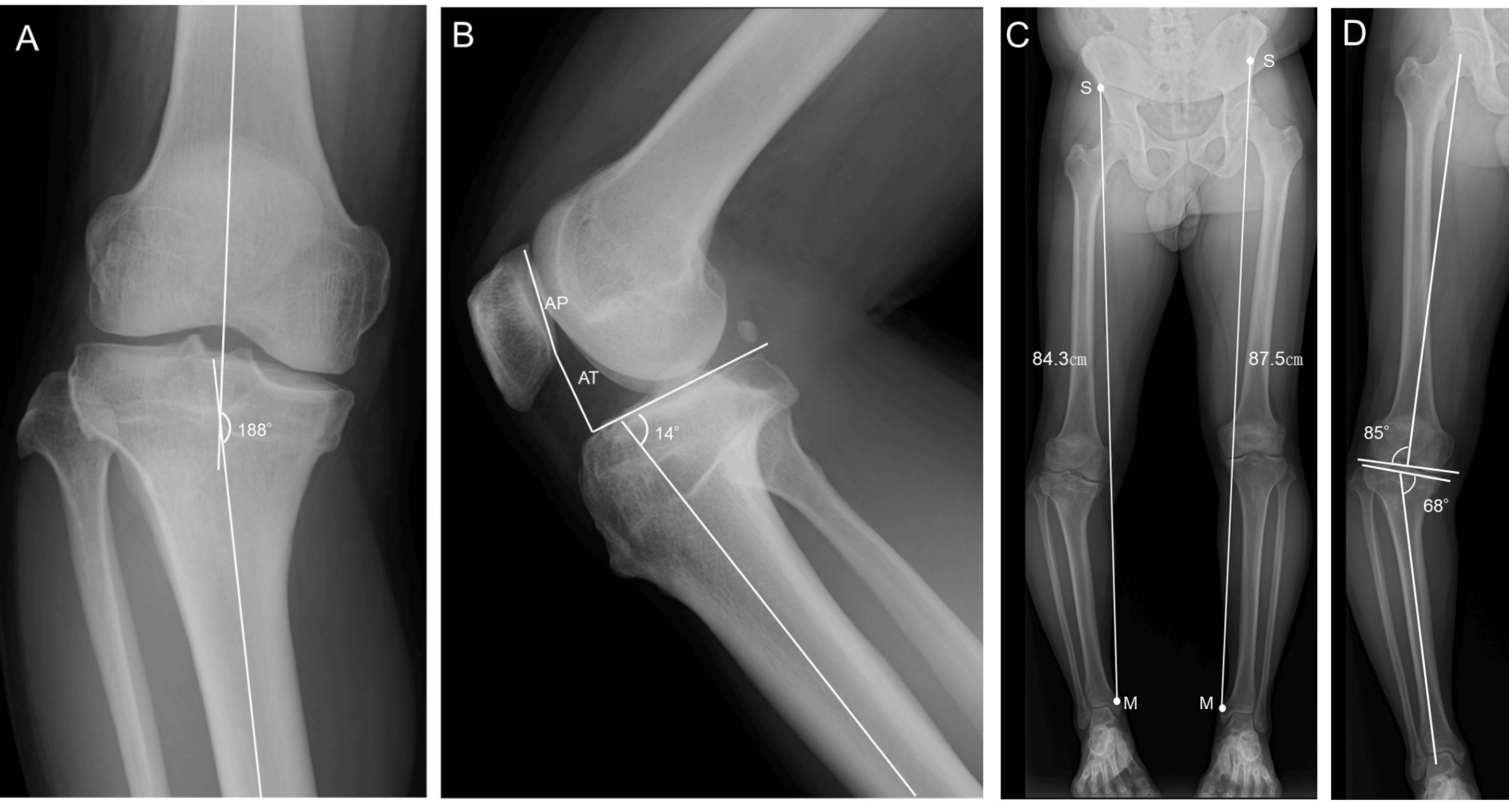
Preoperative radiographs A: Anteroposterior view of the right knee showing a femorotibial angle (FTA) of 188°. B: Lateral view of the right knee showing a Caton–Deschamps index (CDI) of 0.84, calculated as CDI = AT/AP, where AT is the distance between the anterior angle of the tibial plateau and the most inferior aspect of the patellar articular surface, and AP is the patellar articular surface length, as well as a posterior tibial slope of 14°. C: Standing full-length anteroposterior view of both lower limbs showing the spina–malleolar distance (SMD), measured from the anterior superior iliac spine (S) to the medial malleolus (M), which was 84.3 cm on the right and 87.5 cm on the left. D: Standing full-length anteroposterior view of the right lower limb showing a mechanical lateral distal femoral angle (mLDFA) of 85° and a medial proximal tibial angle (MPTA) of 68°.

Radiographs revealed a femorotibial angle (FTA) of 188°, the Caton-Deschamps index (CDI) of 0.84, a posterior tibial slope of 14°, a mechanical lateral distal femoral angle (mLDFA) of 85°, and a medial proximal tibial angle (MPTA) of 68°. The radiographic SMD was 84.3 cm on the right and 87.5 cm on the left (Figure 1).

**Figure 2:**
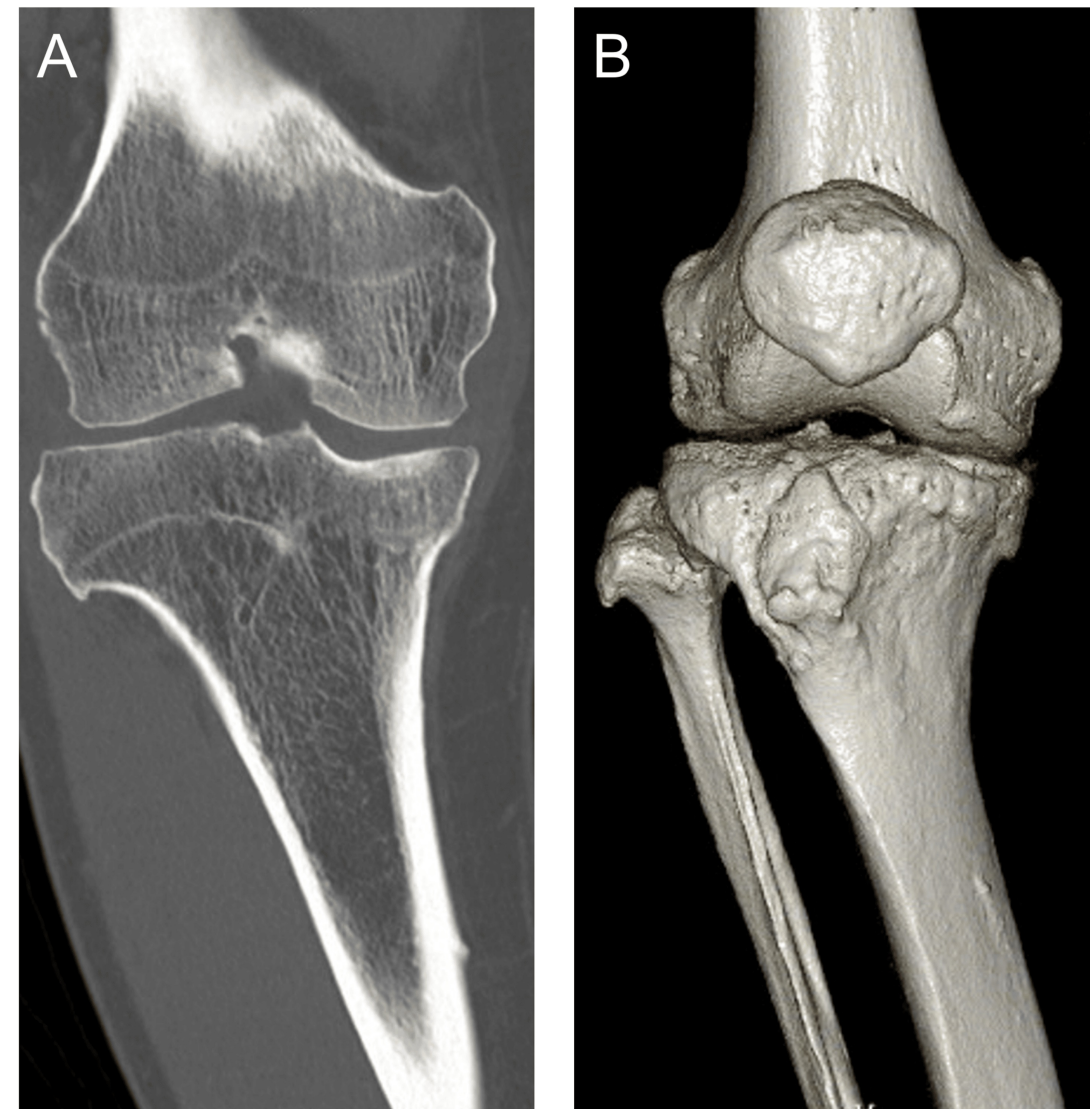
Preoperative CT A: Coronal view of the right knee; B: 3D reconstruction of the right knee

Computed tomography (CT) demonstrated bony union of the prior fracture without osteophyte formation in the lateral or intercondylar regions (Figure 2).

**Figure 3:**
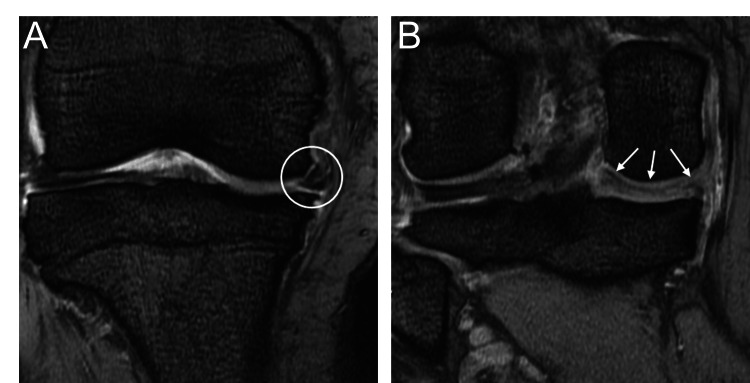
Preoperative MRI A: Medial extrusion of the medial meniscus body (mid-segment) of the right knee; B: Bulging of the medial meniscus posterior horn (posterior segment) of the right knee.

Magnetic resonance imaging (MRI) showed medial displacement of the medial meniscus body and swelling suggestive of a medial meniscus posterior root tear (MMPRT) (Figure 3).

Surgical procedure

Preoperative planning aimed to achieve an MPTA of 88° and a mechanical axis (%MA) of 62%. The procedure was performed under general anesthesia with femoral nerve block. A medial skin incision was made on the lower leg, and the superficial layer of the medial collateral ligament (MCL) was completely detached.

**Figure 4:**
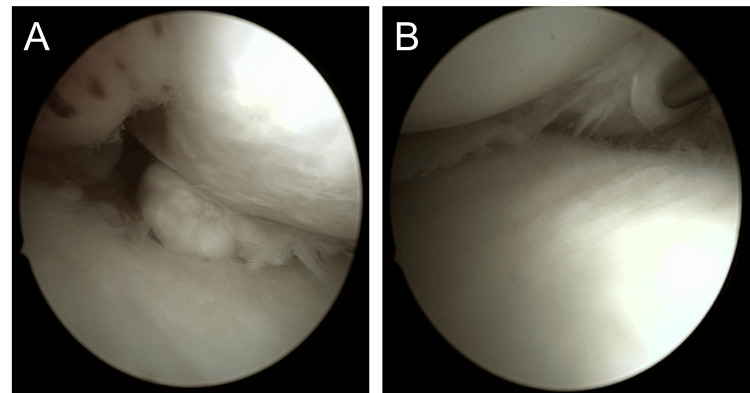
Intraoperative arthroscopic findings A: Medial Meniscal Posterior Root Tear (MMPRT) of the right knee; B: Medial meniscus extrusion of the right knee.

Arthroscopy revealed medial displacement of the mid-portion of the medial meniscus, with exposure of the medial edge of the tibial plateau. A type II MMPRT, according to the LaPrade classification [4], was identified (Figure 4).

**Figure 5:**
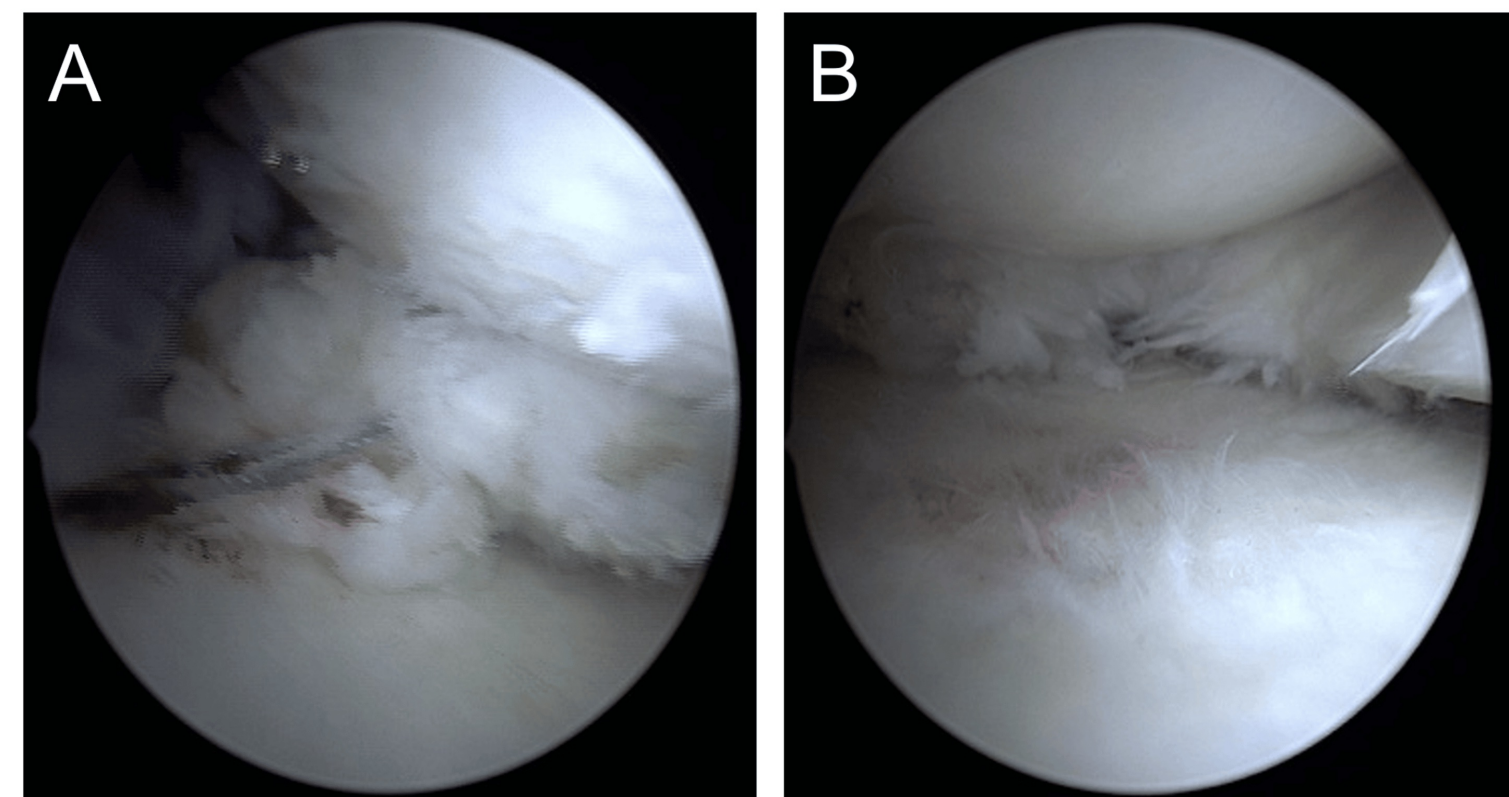
Intraoperative arthroscopic findings A: After anchor fixation for MMPRT (Medial Meniscal Posterior Root Tear); B: After medial meniscus centralization.

Centralization of the medial meniscus and repair of the posterior root tear were performed using suture anchors with the all-inside technique (Figure 5).

Subsequently, osteotomy was performed. Following the method of Akiyama et al. [2], a curved osteotomy was created distal to the tibial tuberosity. A correction angle of 20° with a 25-mm opening gap was achieved. The target correction angle was intraoperatively assessed under fluoroscopic guidance using an angle board to confirm the MPTA. The opening angle was adjusted using a calibrated wedge opener, and the opening width was measured at the lateral cortical hinge using a sterile ruler. Based on the achieved correction angle of 20° and an opening gap of 25 mm, the autologous iliac crest bone graft was trimmed accordingly. The osteotomy site was filled with autologous iliac crest bone graft (block type) combined with granular β-tricalcium phosphate (β-TCP). The osteotomy site was gently compressed, and an additional anterior-to-posterior screw was inserted to enhance stability.

For fixation, a TriS Medial HTO Plate System (Purple, Olympus Terumo Biomaterials Corp., Tokyo, Japan) was applied medially, along with an additional small plate placed anteromedially through the same incision to create a double-plate construct.

Postoperative course

Postoperatively, external immobilization was applied. ROM exercises were initiated at postoperative week two within a range of 0-90°. At week three, one-third partial weight bearing (PWB) was started. By week four, ROM was increased to 0-120° with half PWB, and from week six, full weight bearing (FWB) and unrestricted ROM were permitted. At final follow-up, knee ROM had improved compared with the preoperative status, from 130° of flexion and −15° of extension preoperatively to 140° of flexion and full extension (0°) at five months postoperatively.

**Figure 6:**
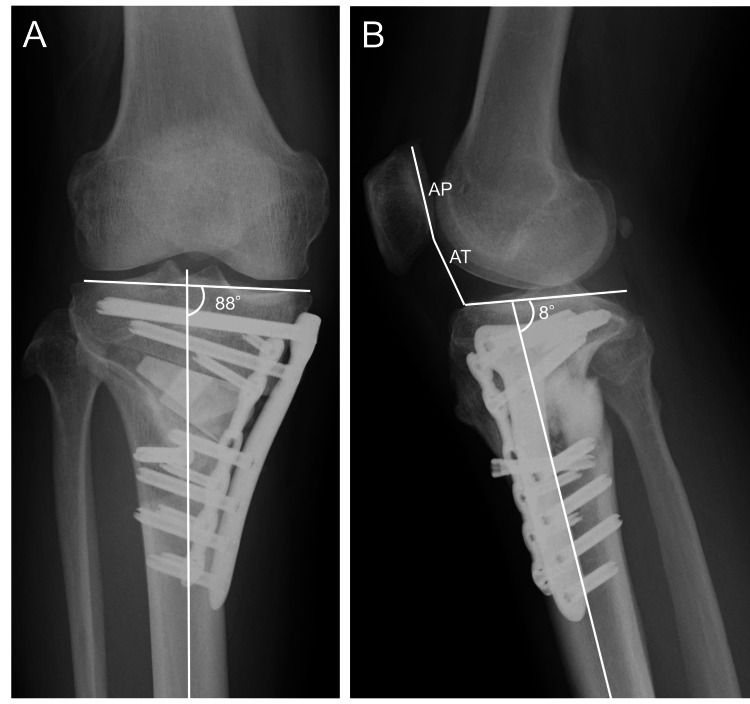
Postoperative radiographs at one month A: Anteroposterior view of the right knee showing a medial proximal tibial angle (MPTA) of 88°; B: Lateral view of the right knee showing a Caton–Deschamps index (CDI) of 0.83 and a posterior tibial slope of 8°.

Postoperative radiographs at one month demonstrated satisfactory alignment, with a MPTA of 88°, preservation of patellar height (CDI of 0.83), and a posterior tibial slope of 8° (Figure 6).

**Figure 7:**
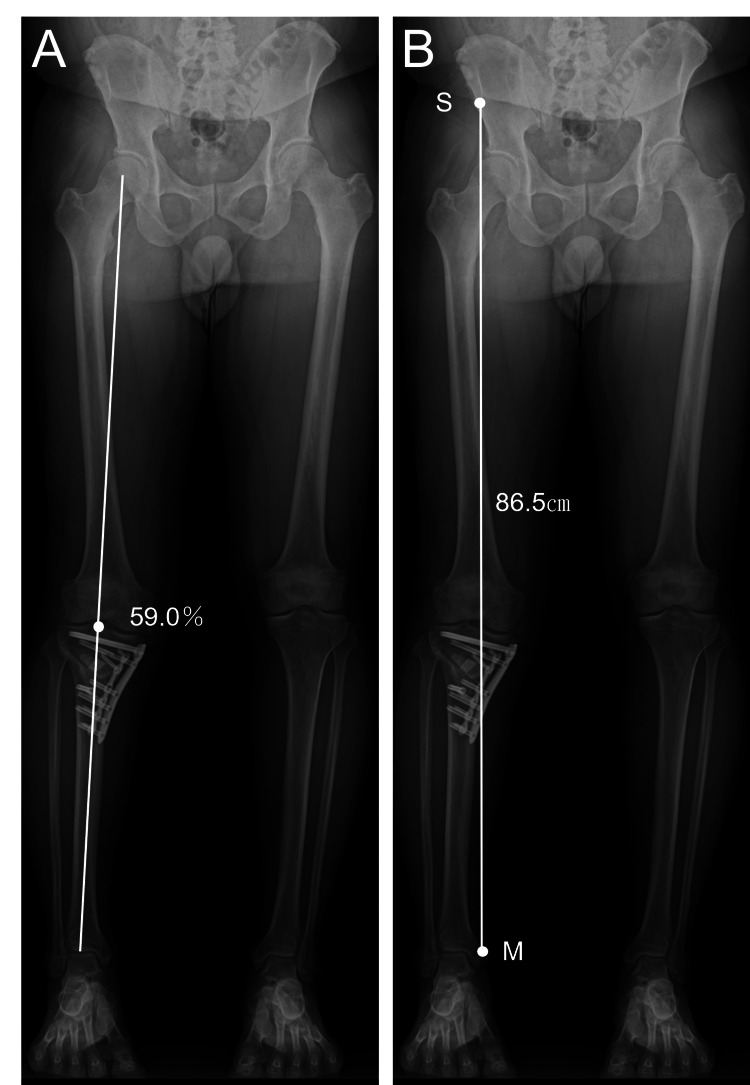
Postoperative standing full-length anteroposterior radiographs of both lower limbs at two months A: Radiograph demonstrating a weight-bearing line ratio (%WBL) of 59.0%; B: Radiograph demonstrating the spina–malleolar distance (SMD) of 86.5 cm. The landmarks for measurement are indicated as S (anterior superior iliac spine) and M (medial malleolus), as shown in Figure 1.

At two months postoperatively, standing full-length radiographs showed a weight-bearing line ratio (%WBL) of 59.0% and a radiographic SMD of 86.5 cm (Figure 7).

Although the radiographic appearance suggested a slight residual limb length difference, both radiographic and clinical (surface-measured) SMD demonstrated an approximately 1 cm discrepancy between sides, which was considered clinically acceptable. Minor discrepancies between radiographic and surface measurements may be influenced by limb positioning and knee extension during imaging.

**Figure 8:**
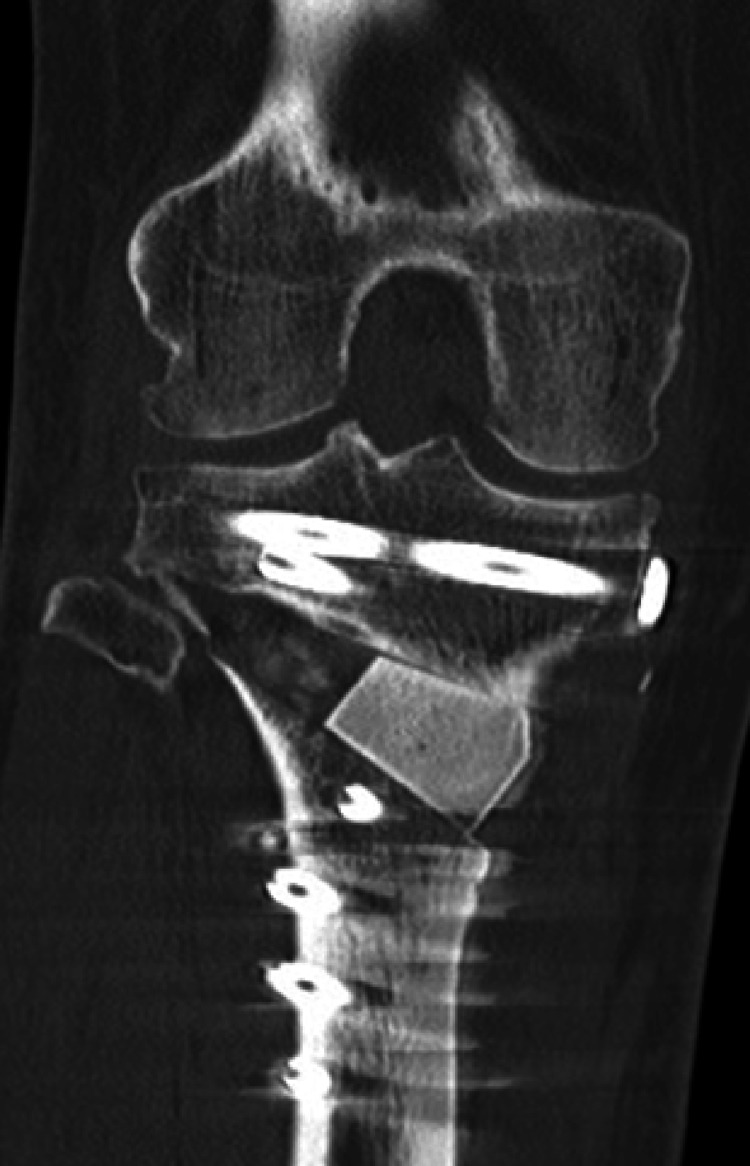
Postoperative CT at one month Coronal CT view of the right knee showing the open wedge site filled with the autologous bone graft and fixation with a locking plate.

**Figure 9:**
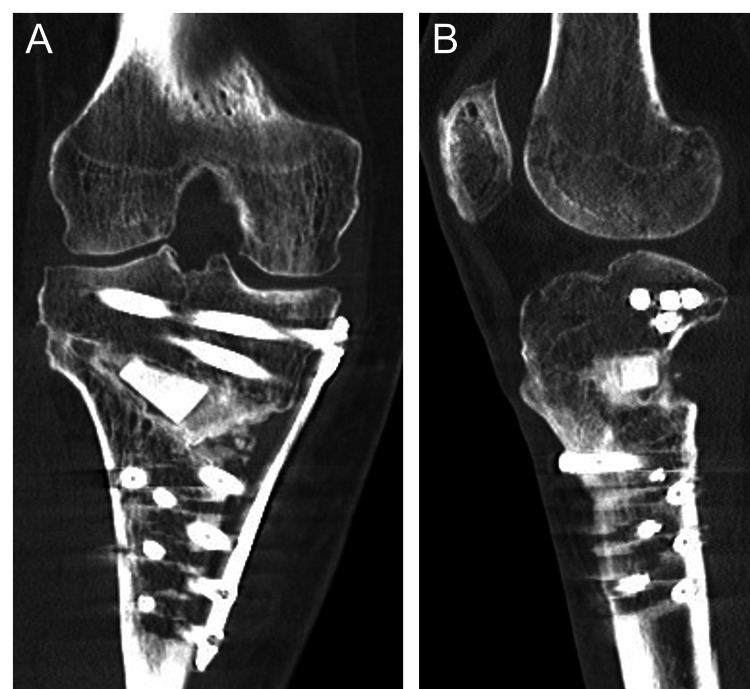
Postoperative CT at seven months A: Coronal CT view of the right knee; B: Sagittal CT view of the right knee.

CT at one month postoperatively revealed a type I hinge fracture according to the Takeuchi classification (Figure 8); however, no correction loss was observed during follow-up. CT at seven months demonstrated progressive bone healing, particularly in the anterior and medial aspects of the osteotomy site (Figure 9).

## Discussion

This case presented several technical considerations in achieving optimal alignment correction while ensuring stable fixation and maintaining patellar height.

A major preoperative concern was an LLD of approximately 30 mm. An LLD greater than 10 mm may lead to gait disturbance, pelvic tilt, and secondary spinal deformity [5]. In OWHTO, the operated limb is lengthened proportionally to the correction angle. He et al. [6] reported a mean lengthening of 7.5 ± 2.3 mm with an average correction of 11.7° ± 4.6°, whereas Kim et al. [7] found that subjective awareness of LLD was more frequent after OWHTO than after CWHTO. Considering the patient’s age (54 years), BMI (28.6 kg/m²), and preexisting 32 mm discrepancy, both varus correction and leg length equalization were prioritized.

**Figure 10:**
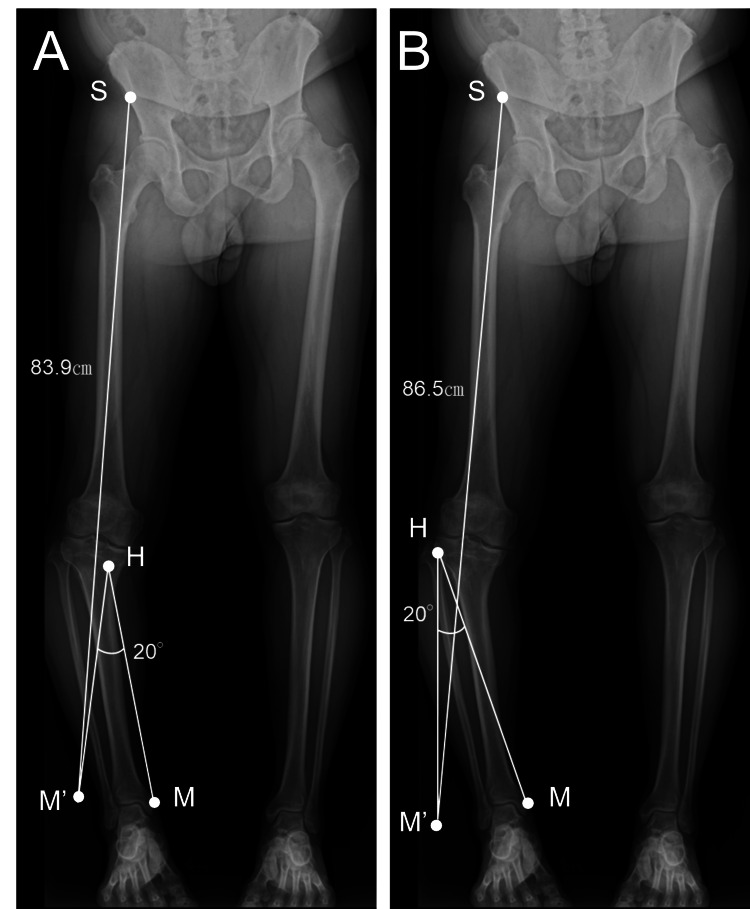
Preoperative simulation comparing open-wedge distal tuberosity tibial osteotomy (OWDTO) and hybrid closed-wedge high tibial osteotomy (HCWHTO) for prediction of postoperative spina–malleolar distance (SMD). A: Hybrid closed-wedge high tibial osteotomy (HCWHTO). The osteotomy was initiated approximately 40 mm distal to the lateral tibial cortex and extended medially to approximately 15 mm distal to the joint line. The hinge point (H) was defined near the joint surface at the medial one-third of the line connecting the lateral starting point and the medial endpoint. A simulated 20° correction around the hinge point displaced the medial malleolus from its pre-correction position (M) to the post-correction position (M′), resulting in a predicted postoperative SMD of 83.9 cm. The anterior superior iliac spine is indicated as (S). B: Open-wedge distal tuberosity tibial osteotomy (OWDTO). The osteotomy was initiated approximately 35 mm distal to the medial tibial joint line and directed toward the tip of the fibular head, with the hinge point (H) set 5 mm medial to the lateral tibial cortex. A simulated 20° correction around the hinge point displaced the medial malleolus from (M) to (M′), resulting in a predicted postoperative SMD of 86.5 cm. The anterior superior iliac spine is indicated as (S). In both simulations, postoperative SMD was calculated as the linear distance between the anterior superior iliac spine (S) and the medial malleolus after correction (M′).

Preoperative simulation comparing OWDTO and HCWHTO predicted postoperative SMDs of 86.5 cm and 83.9 cm, respectively (Figure 10).

In the OWDTO simulation, the osteotomy was initiated approximately 35 mm distal to the medial tibial joint line and directed toward the tip of the fibular head, with the hinge point intentionally set 5 mm medial to the lateral tibial cortex to preserve lateral cortical continuity. In contrast, in the HCWHTO simulation, the osteotomy was initiated approximately 40 mm distal to the lateral tibial cortex and extended medially to approximately 15 mm distal to the joint line. The hinge point was defined near the joint surface at the medial one-third of the line connecting the lateral starting point and the medial endpoint, reflecting the characteristic geometry of the hybrid closed-wedge technique. In both simulations, a correction angle of 20° was applied around the defined hinge point, and the predicted postoperative SMD was calculated based on the displacement of the medial malleolus from its pre-correction position (M) to the post-correction position (M′). Because OWDTO was expected to provide better correction with improved limb length symmetry, it was selected. Intraoperatively, angular correction was assessed under fluoroscopic guidance using an angle board to confirm the medial proximal tibial angle, while the opening angle and width were adjusted using a calibrated wedge opener and direct measurement at the lateral cortical hinge, ensuring reproducibility of the planned correction. It should be noted that radiographic SMD measurements may be influenced by patient positioning, limb rotation, and radiographic technique, and therefore may not correspond exactly to surface measurements. In the present case, postoperative radiographic SMD was consistent with the preoperative simulation, demonstrating a residual limb length difference of approximately 1 cm. This degree of discrepancy was confirmed by both radiographic and clinical assessments and was considered clinically acceptable.

Another important consideration was the risk of delayed bone union due to the large correction angle (20°) and opening gap (25 mm). Shiboni et al. [8] identified a correction angle >10° as a strong predictor of nonunion (odds ratio 10.5), and Araya et al. [9] reported that an opening width >10.5 mm increased the risk of delayed union. To promote early healing, autologous iliac bone grafting was performed prophylactically. Nicholas et al. [10] reported a nonunion rate of only 0.5% in cases with bone grafting, and Bodenbeck et al. [11] demonstrated a 100% union rate within 12 months with grafting compared with 60% without. In this case, CT at seven months postoperatively confirmed progressive bone formation, particularly in the anterior and medial regions, suggesting that grafting contributed to favorable healing.

Given the large correction angle, the potential for a hinge fracture was another concern. According to the Takeuchi classification [12], type I fractures are generally stable and rarely result in correction loss, whereas types II and III are more unstable and should be avoided [13]. To enhance stability, a supplementary small plate was applied to the anteromedial aspect in addition to the medial plate. Although a type I hinge fracture was detected on CT at one month postoperatively, correction was maintained, indicating that the double-plate construct provided sufficient mechanical stability.

Preservation of patellar height, as represented by the CDI, was also an essential factor in determining the osteotomy technique. Conventional OWHTO tends to lower the patella by elevating the tibial tubercle relative to the joint line, potentially increasing patellofemoral pressure. Han et al. [14] reported that OWDTO maintains patellar height and reduces the risk of patellofemoral degeneration. Similarly, Ren et al. [15] showed that OWDTO minimizes changes in CDI compared with standard OWHTO. Because the CDI in this patient was within the normal range preoperatively, maintaining it was essential to prevent anterior knee pain and patellofemoral overload.

Clinically, knee ROM improved from the preoperative state to 140° of flexion and full extension by five months postoperatively, indicating satisfactory functional recovery without stiffness or extension deficit. Although short-term outcomes were favorable, potential long-term risks such as patellofemoral degeneration and altered joint loading should be considered, and continued follow-up is warranted.

In summary, OWDTO with autologous bone grafting and double-plate fixation provided stable correction and reliable union while preserving patellar height and improving limb alignment. This technique may be particularly advantageous for severe post-traumatic varus deformities requiring large angular correction and restoration of limb symmetry.

## Conclusions

This case demonstrated that the combination of OWDTO, autologous bone grafting, and double-plate fixation is an effective strategy for correcting severe post-traumatic varus knee deformity accompanied by leg length discrepancy. Careful preoperative planning, including simulation of limb length changes and consideration of patellar height preservation, is essential when selecting the optimal osteotomy technique in cases requiring large angular correction. This combined approach provided stable fixation, promoted reliable bone union, preserved patellar height, and achieved satisfactory functional recovery, suggesting that it represents a practical and reproducible option for complex deformities with substantial correction demands.
